# Knowledge and management of chronic spontaneous urticaria in Latin America: a cross-sectional study in Ecuador

**DOI:** 10.1186/s40413-017-0150-7

**Published:** 2017-05-23

**Authors:** A. Cherrez, M. Maurer, K. Weller, J. C. Calderon, D. Simancas-Racines, I. Cherrez Ojeda

**Affiliations:** 1 Respiralab Research Group, Respiralab, Guayaquil, Ecuador; 2 School of Medicine, University of Heidelberg, Heidelberg, Germany; 3 Department of Dermatology and Allergy, Allergie-Centrum-Charité, Charité – Universitätsmedizin Berlin, Berlin, Germany; 4 School of Medicine, Universidad Espiritu Santo, Samborondón, Guayas Ecuador; 5 Centro de Investigación en Salud Pública y Epidemiologia Clínica, Facultad de Ciencias de la Salud Eugenio Espejo, Universidad Tecnológica Equinoccial, Quito, Ecuador

**Keywords:** Chronic urticaria, Guidelines, Latin America, Management, Treatment

## Abstract

**Background:**

The current EAACI/GA^2^LEN/EDF/WAO guideline for urticaria provide specific recommendations for the diagnostic workup and treatment of patients with chronic spontaneous urticaria (CsU). This study explored if physicians in Ecuador know these recommendations and implement them in their actual clinical practice for CsU.

**Methods:**

We investigated physicians who treat CsU patients in a cross-sectional study using a standardized questionnaire. Descriptive statistics were employed, adjusted logistic regression was performed to assess the link of guideline knowledge and use of therapy.

**Results:**

Seven hundred forty surveys were collected and analyzed. The mean age of physicians was 42.3 (±12.5) years. Most of the participants (65.1%) were general physicians (GP), 13.7% were pediatricians, 11.0% internists, 6.8% dermatologists or allergists (D/A). Only 18.8% knew the EAACI/GA^2^LEN/EDF/WAO guideline. 44.5% of GPs searched for CsU etiology in contrast to 90% of D/A. Most common diagnostic test was total serum IgE (83.5%). Most common first line symptomatic treatment was oral corticoids (46.3%), followed by second generation antihistamines (sgAHs, 36.8%). A/D prescribed more sgAHs (regular doses) (74.1 vs 28.6% of GP) (*p* < 0.05). Experience with omalizumab was reported only by 3.5%, of physicians, and higher rates among who were familiar with the guideline.

**Conclusion:**

This study shows that the knowledge of guideline recommendations in physicians who treat urticaria patients in Ecuador is low. The diagnostic workup and treatment of CsU patients are largely not in line with guideline recommendations in real life practice settings. We were able to compare results between German and Ecuadorian physicians and found that Ecuadorian physicians have lower awareness of the current guideline (33 vs 18%). Only one-third of physicians reported using regular doses of sgAHs as the first line treatment. Also, only 12.9% of physicians use sgAHs in higher doses and physicians still use fgAHs, particularly pediatricians (42.9%). Our results suggest that disparities in knowledge between physicians from different countries could influence the management of CsU. Knowledge of the guidelines is linked to better choices of treatments. Awareness of guidelines needs to be promoted for better management of chronic urticaria.

**Electronic supplementary material:**

The online version of this article (doi:10.1186/s40413-017-0150-7) contains supplementary material, which is available to authorized users.

## Background

Urticaria is a disease characterized by the development of wheals (hives), angioedema or both. Chronic urticaria (CU) is defined by the appearance of these signs and symptoms for ≥6 weeks and is categorized into two main types: chronic spontaneous urticaria (CsU) and chronic inducible urticaria [1]. CsU is thought to affect 0.5–1% of the global population at any given time, accounting for approximately two-thirds of all cases of CsU. Chronic Urticaria can have a considerable burden on patients, healthcare systems and society [2].

Different studies have evaluated the economic impact of CsU and a total annual cost of $ 2047 per patient has been estimated, with indirect costs accounted for 15.7% ($322) [3]. More importantly, this disease is commonly associated with an impairment of patients in many aspects of their daily living (e.g. their choice of clothes or food) [4], with marked impact on their quality of life (QoL) and productivity (e.g. impaired work performance, absence from work) [3].

Several guidelines, consensus papers, and practice parameters are available for the management of chronic urticaria. The leading international guideline is the EAACI/GA^2^LEN/EDF/WAO guideline for urticaria, which was revised and updated in 2013. National practice parameters include those of the US American AAAAI/ACAAI Joint Task Force, which were updated in 2014. The recommendations given by all of these documents are similar, although some minor differences exist [5]. For example, the EAACI/GA^2^LEN/EDF/WAO guideline for urticaria does not recommend H2 antihistamines or first generation antihistamines for CsU, whereas the US practice parameters do [6].

The EAACI/GA^2^LEN/EDF/WAO guideline for urticaria provides a set of specific recommendations for the management of CU. For example, they recommend a stepwise approach in the management of CsU, beginning with licensed doses of modern second generation H1-antihistamines (sgAHs) [1] as the first line therapy, which is effective in resolving symptoms in about 40% of patients with CsU [2]. As second line therapy, the guideline recommends increasing the doses of sgAHs up to four times the regular doses, if a patient does not respond to first line treatment after 2 weeks. For patients non-responsive to higher doses of sgAHs, the guideline recommends the addition of a third-line treatment option such as omalizumab, cyclosporine or montelukast [1].

Recent studies on guideline and awareness in physicians who treat urticaria patients have shown interesting results. In one such study, performed in Germany, the level of urticaria guideline knowledge was highest in dermatologists (50.6%) when compared with pediatricians (24.2%) and general physicians (12.6%). Physicians who were familiar with the guidelines were significantly more likely to perform useful diagnostic tests such as the ASST (autologous serum skin test). However, even in physicians who knew the guideline, the test was still only performed by one of five physicians. Physicians who stated to be familiar with the guidelines were less likely to use sedating antihistamines and systemic steroids as a first- and/or second-line treatment, indicating that guideline recommendations may improve the quality of care [7].

Most of the studies performed indicate that the knowledge and awareness of guidelines for the management of patients with chronic urticaria is low in physicians who treat them. This may be responsible, at least in part, for the fact that more than 70% of the patients had stopped consulting a physician, resigning themselves to self-treatment or simply living with their condition [8].

As of now, little is known about the knowledge of urticaria guidelines in physicians in Latin America who treat urticaria patients or about their clinical practice and the impact of guidelines. Our study intended to explore the awareness and knowledge of urticaria guidelines in physicians in Ecuador and to better characterize and understand the actual clinical practice for CsU.

## Methods

From March 2015 to March 2016 we conducted a cross-sectional survey study using a standardized questionnaire. This was approved by the Ethics Committee of Hospital Luis Vernaza.

The participants were physicians, and this survey covered several topics about CsU: general questions about urticaria (e.g. prevalence, duration, and disease activity), questions about the diagnostic and therapeutic management of CsU as well as consequences of insufficient symptom control. In addition, questions on how the physicians perceive the patients, self-assessment of the participating physicians and questions concerning the implementation of the current guidelines were also addressed.

### Recruitment

The target populations were general physicians and specialists from Ecuador attending the Respiratory and Allergy Medicine Conference and physicians working in hospitals and private practices in different cities of Ecuador. The participation criterion was having a diploma in medicine and being a certified medical doctor. When a candidate was identified, we asked if he/she often sees patients with urticaria. Once agreed, the data collection team asked for consent and delivered the self-assessment survey.

### Study survey

The original survey questions were previously developed by an expert panel from Germany. It has been successfully pretested for comprehensibility and feasibility in a limited number of physicians (*n* = 32). We used a rigorous method of validation for the translated version of the German questionnaire [1, 9] which we briefly described. Two official translators translated the German version (GV) of the original survey to Spanish. Next, the Spanish-language version was translated to German language by a third translator who did not know the original version of the questionnaire. Then, the back-translated German version of the new Spanish-language questionnaire was compared with the original German-language version. Each item on the back-translated German-language version was ranked by 30 individuals who were bilingual and independent of the study team for comparability and similarity of interpretability with the same item on the original German-language version. Any translated item with a mean score >3 (seven was the worst agreement and one was the best agreement) was formally reviewed and corrected. The revised item was then translated back to German and compared again with the original German-language version of that item. This process continued until the mean scores for each item indicated a valid version (<3 on each of the comparability and interpretability rankings, and preferably <2.5 on the interpretability rankings) [9].

In total, the survey consisted of 32 questions, mostly involving Likert-scale ratings, quantitative questions, yes ⁄ no lists and multiple choice questions (the complete survey can be provided upon request). Only physicians who reported attending at least one patient at month with idiopathic chronic urticaria were considered for analysis. Also, the outliers values of patients reported to be attended with chronic urticaria at last month were excluded of analysis (*n* = 31, 6.6%). After that, the values left blank automatically were treated as missing values, in total, 7.3% of data were missed. Further missing value analysis determined that these missing values were random.

### Statistical analysis

Descriptive statistics were employed to compare frequencies and percentages values are given as means and standard deviation (SD), unless specified otherwise. We used Chi square test to compare proportions across specialty groups (allergists/dermatologist, general physician, internal medicine, pediatrician, and others) and between physicians who do or do not know the EAACI/GA^2^LEN/EDF/WAO guideline for the management of urticaria. Finally, adjusted logistic regression was performed to test the association of guideline knowledge and use of second line therapy for confounders (physicians’ specialty and location, years in practice, and gender. SPSS version 20 (SPSS, Inc, Chicago, IL) was employed. A p value of less than 0.05 was considered significant for all tests.

## Results

### Demographics

From March 2015 to March 2016, a total of 740 surveys were collected, and 438 (59.2%) of physicians reported attending at least one patient with idiopathic chronic urticaria. The mean age of the physicians was 42.3 (±12.5) years, an average of 15.0 years of practice (±11.2), and 51.5% were female. The majority of the participants (65.1%) were general physicians (GP), 13.7% were pediatricians, 11.0% internists, 6.8% dermatologists or allergists (D/A), and 3.4% belonged to other professional groups (Table 1). Most of the physicians (81.1%) worked in urban areas and 47.0% were working as single practice physicians. Pediatricians reported to see 5.3 (SD 5.1) patients with CsU compared to 15.8 (SD 9.6) patients with atopic dermatitis per month.

**Table 1:** Demographic data according to specialties

	GPs		A/D		Others		Ped		IM		Total	
	*n*	%	*n*	%	*n*	%	*n*	%	*n*	%	*n*	%
Age, mean - SD	38.6	12.1	45.9	9.3	48.7	10.5	50.0	10.3	50.1	10.4	42.3	12.5
Male	132	48.2	12	40.0	9	69.2	24	42.1	27	57.4	204	48.5
Female	142	51.8	18	60.0	4	30.8	33	57.9	20	42.6	217	51.5
Less than 20 years in practice	207	73.4	15	50.0	6	40.0	19	31.7	18	37.5	265	60.9
Between 20 to 30 years in practice	51	18.1	12	40.0	6	40.0	36	60.0	19	39.6	124	28.5
More than 30 years in practice	24	8.5	3	10.0	3	20.0	5	8.3	11	22.9	46	10.6
Rural	73	25.8	1	3.3	0	0.0	4	6.7	4	8.5	82	18.9
Urban	210	74.2	29	96.7	15	100.0	56	93.3	43	91.5	353	81.1

*GPs* General Physician, *A/D* Allergist/Dermatologists, *Ped* Pediatricians, *IM* Internal Medicine

### Few physicians know the EAACI/GA^2^LEN/EDF/WAO urticaria guideline

Only 79 of 421 (18.8%) physicians reported to know the EAACI/GA^2^LEN/EDF/WAO urticaria guideline, and more than half of them (66.7%) were D/As (Additional file 1: Figure S1). 60 of 419 (14.3%) physicians surveyed reported that they know other guidelines (Additional file 2: Figure S2).

### Chronic spontaneous urticaria is a frequent diagnosis

In general, physicians attended a mean of 4.7 patients with CsU per month (SD 4.4, median 3.0, IQR 2 – 6). GPs responded that they see approximately 4.8 patients with CsU per month (SD 4.5). D/As reported that they see 4.3 (SD 3.1) patients with CsU per month, as compared to 5.2 (SD 2.8) patients with psoriasis vulgaris. In contrast, pediatricians reported to see 5.3 (SD 5.1) patients with CsU compared to 15.8 (SD 9.6) patients with atopic dermatitis per month.

### CsU patients are subjected to multiple diagnostic tests

**Fig. 1:**
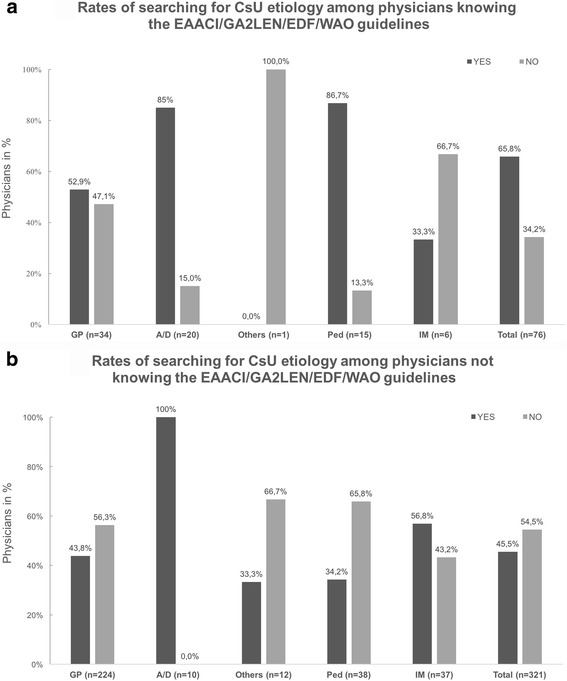
**a** Rates of searching for csU etiology according to specialty among physicians who know the EAACI/GA2LEN/EDF/WAO guidelines, in percentage. **b** Rates of searching for csU etiology according to specialty among physicians who don’t know the EAACI/GA2LEN/EDF/WAO guidelines, in percentage

196 physicians (49.4%) reported to look for causes of CU. 52.9% of the GPs search for the causes of CsU in contrast to 85.0% of the D/As who were aware of guidelines (Fig. 1 a–b). The cause of urticaria was identified only in approx. 34.2% of patients and this was similar for both GPs and D/As.

The most common diagnostic tests performed were total serum IgE (83.5%), differential blood count (59.5%), serological tests (56.5%), allergy test (prick test) (56.0%), C-reactive protein ⁄ erythrocyte sedimentation rate (46.5%), autoimmune diagnostic (45.5%), thyroid hormones and autoantibodies (34.5%), and autologous serum skin test (13.5%).

Important differences were observed in the preference for laboratory testing and other specialist consultations between groups. The majority of the D/As ordered more serological tests in comparison to the GPs (Table 2), and there was a similar trend for the diagnostic workup of autoimmune diseases, thyroid hormones and autoantibodies, IgE tests, helicobacter tests, dentist consultation (*p* <0.05). However, autologous serum skin test and allergy test (prick test) were similar between all groups (Table 2). Diagnostic tests were performed more frequently by physicians who know the EAACI/GA^2^LEN/EDF/WAO guideline (Table 2).

**Table 2:** Diagnostic tests for searching CsU etiology and knowledge of the EAACI/GA^2^LEN/EDF/WAO guideline according to specialties

	GPs		A/D		Others		Peds		IM		Total		Chi square	Aware of guideline		Not aware of guideline		Total		Chi square
	*n*	%	*n*	%	*n*	%	*n*	%	*n*	%	*n*	%	*p* value	*n*	%	*n*	%	*n*	%	*p* value
Differential count of leukocytes	64	55.2	20	74.1	3	60.0	17	60.7	15	62.5	119	59.5	0.495	33	66.0	83	57.2	116	59.5	0.277
Serological tests	56	48.3	23	85.2	3	60.0	17	60.7	14	58.3	113	56.5	*0.014*	30	60.0	80	55.2	110	56.4	0.553
CRP/ESR	49	42.2	18	66.7	2	40.0	13	46.4	11	45.8	93	46.5	0.253	35	70.0	55	37.9	90	46.2	*0.000*
C1 esterase inhibitor	6	5.2	1	3.7	0	0.0	2	7.1	2	8.3	11	5.5	0.910	3	6.0	8	5.5	11	5.6	0.898
Autoimmune disease diagnoses (eg: ANA)	45	38.8	19	70.4	0	0.0	14	50.0	13	54.2	91	45.5	*0.007*	27	54.0	64	44.1	91	46.7	0.228
Autologous serum skin test	13	11.2	4	14.8	0	0.0	4	14.3	6	25.0	27	13.5	0.396	10	20.0	17	11.7	27	13.8	0.144
Microbiology analyses	17	14.7	18	66.7	1	20.0	7	25.0	7	29.2	50	25.0	*0.000*	18	36.0	31	21.4	49	25.1	*0.040*
Auto-antibodies and thyroid hormones	38	32.8	19	70.4	0	0.0	5	17.9	7	29.2	69	34.5	*0.000*	23	46.0	44	30.3	67	34.4	*0.044*
Allergy test (eg: Prick test)	62	53.4	13	48.1	2	40.0	20	71.4	15	62.5	112	56.0	0.329	34	68.0	77	53.1	111	56.9	0.067
IgE levels	97	83.6	26	96.3	2	40.0	22	78.6	20	83.3	167	83.5	*0.032*	46	92.0	117	80.7	163	83.6	0.063
Low intake of pseudo-allergens	49	42.2	17	63.0	1	20.0	9	32.1	7	29.2	83	41.5	0.071	29	58.0	54	37.2	83	42.6	*0.010*
Helicobacter test	25	21.6	16	59.3	0	0.0	11	39.3	6	25.0	58	29.0	*0.001*	19	38.0	37	25.5	56	28.7	0.093
Dentistry consultation	2	1.7	4	14.8	0	0.0	0	0.0	1	4.2	7	3.5	*0.014*	3	6.0	4	2.8	7	3.6	0.288
Instrumental test (eg: ultrasound)	12	10.3	4	14.8	0	0.0	7	25.0	4	16.7	27	13.5	0.269	6	12.0	21	14.5	27	13.8	0.661
ENT consultation	8	6.9	7	25.9	1	20.0	2	7.1	4	16.7	22	11.0	*0.045*	6	12.0	15	10.3	21	10.8	0,745
Others	2	1.7	2	7.4	0	0.0	1	3.6	3	12.5	8	4.0	0.130	1	2.0	6	4.1	7	3.6	0.483

*CRP* C-reactive Protein, *ESR* erythrocyte sedimentation rate

### Most physicians do not treat CsU patients according to guideline recommendations

The most used first choice symptomatic treatment in previously untreated patients was an oral corticosteroid (OCS), by 46.3% of physicians. 40.7% of D/As reported having experience with OCS treatment, but only 57.1% are convinced that this therapy is useful. Second generation antihistamines (sgAHs) were used in approved dose by 36.8% of physicians as first line treatment; (74.1%) of D/As, (29.1%) of GPs (28.6% of pediatricians and 37.5% of internists, (*p* < 0.001, Table 3). First generation antihistamines (fgAHs), at normal doses, were used by 13.9% of the respondents as first choice treatment. Meanwhile, fgAHs at higher doses were used by 7.0%. Physicians, who were familiar with the EAACI/GA^2^LEN/EDF/WAO guideline, were more likely to use higher doses of fgAHs (14.0 vs 4.8% of those who do not know the guidelines) (*p* < 0.05, Table 3). Despite years of practice, specialties, location, and gender, these physicians report using higher doses of fgAHs (OR 4.3; CI 1.1–16.4) (Table 4). Pediatricians use most frequently (42.9%) fgAHs as first line treatment (Table 3). We did not find any statistical differences (*p* > 0.05) according to awareness of guidelines and use of normal doses of sgAHs (Table 3).

**Table 3:** First line symptomatic treatment and knowledge of the EAACI/GA^2^LEN/EDF/WAO guideline according to specialties

	GPs		A/D		Others		Peds		IM		Total		Chi square	Aware of guideline		Not aware of guideline		Chi square
	*n*	%	*n*	%	*n*	%	*n*	%	*n*	%	*n*	%	*p* value	*n*	%	*n*	%	*p* value
fgAHs (normal doses)	9	7.7	1	3.7	1	20.0	12	42.9	5	20.8	28	13.9	*0.000*	5	10.0	22	15.1	0.369
fgAHs (highest doses)	9	7.7	2	7.4	0	0.0	3	10.7	0	0.0	14	7.0	0.578	7	14.0	7	4.8	0.029
sgAHs (normal doses)	34	29.1	20	74.1	3	60.0	8	28.6	9	37.5	74	36.8	*0.000*	23	46.0	50	34.2	0.138
sgAHs (highest doses)	9	7.7	14	51.9	0	0.0	3	10.7	0	0.0	26	12.9	*0.000*	14	28.0	12	8.2	0.000
Combination of fgAHs	2	1.7	0	0.0	0	0.0	1	3.6	1	4.2	4	2.0	0.802	1	2.0	2	1.4	0.754
Combination of sgAHs	24	20.5	1	3.7	0	0.0	0	0.0	7	29.2	32	15.9	*0.007*	2	4.0	30	20.5	0.006
fgAHs plus sgAHS	5	4.3	1	3.7	0	0.0	0	0.0	0	0.0	6	3.0	0.650	3	6.0	3	2.1	0.162
fgAHs plus AH2	2	1.7	2	7.4	0	0.0	0	0.0	0	0.0	4	2.0	0.261	1	2.0	3	2.1	0.981
sgAHS plus AH2	14	12.0	2	7.4	0	0.0	0	0.0	3	12.5	19	9.5	0.319	1	2.0	18	12.3	0.033
fgAHs plus alk	4	3.4	1	3.7	0	0.0	1	3.6	2	8.3	8	4.0	0.825	3	6.0	4	2.7	0.284
sgAHs plus alk	6	5.1	6	22.2	0	0.0	2	7.1	1	4.2	15	7.5	*0.037*	9	18.0	6	4.1	0.001
fgAHs plus AH2 plus alk	2	1.7	0	0.0	0	0.0	1	3.6	0	0.0	3	1.5	0.789	0	0.0	3	2.1	0.307
sgAHs plus AH2 plus alk	7	6.0	7	25.9	0	0.0	2	7.1	0	0.0	16	8.0	*0.005*	9	18.0	7	4.8	0.003
Ciclosporine	8	6.8	2	7.4	0	0.0	2	7.1	1	4.2	13	6.5	0.958	3	6.0	8	5.5	0.890
Dapsone	3	2.6	7	25.9	0	0.0	2	7.1	2	8.3	14	7.0	*0.001*	8	16.0	6	4.1	0.005
Hydroxychloroquine	1	0.9	3	11.1	0	0.0	1	3.6	0	0.0	5	2.5	0.034	2	4.0	3	2.1	0.451
Ketotifen	2	1.7	1	3.7	0	0.0	0	0.0	1	4.2	4	2.0	0.789	1	2.0	2	1.4	0.754
Methotrexate	3	2.6	6	22.2	0	0.0	0	0.0	0	0.0	9	4.5	*0.000*	8	16.0	1	0.7	0.000
Omalizumab	3	2.6	4	14.8	0	0.0	0	0.0	0	0.0	7	3.5	0.013	6	12.0	1	0.7	0.000
Oral cortico-steroids	53	45.3	11	40.7	2	40.0	13	46.4	14	58.3	93	46.3	0.761	20	40.0	72	49.3	0.255
Sulfasalazine	0	0.0	0	0.0	0	0.0	0	0.0	1	4.2	1	0.5	0.116	0	0.0	1	0.7	0.557
Tricyclic antidepressants	1	0.9	0	0.0	0	0.0	0	0.0	0	0.0	1	0.5	0.949	0	0.0	1	0.7	0.557
Others	0	0.0	6	22.2	1	20.0	1	3.6	0	0.0	8	4.0	*0.000*	3	6.0	5	3.4	0.427

*fgAHs* first generation antihistamines, *sgAHs* second generation antihistamines, *AH-2* anti-H2, *ALK* anti-leukotriene

**Table 4:** Adjusted logistic regression (OR) for predicting prescription of fgAHs (higher doses) and specialty (reference: internal medicine), years of practice (reference: <20 years in practice), location (reference: rural), gender (reference: female), and awareness of EAACI/GA^2^LEN/EDF/WAO Guidelines (reference: no)

	*p* value	OR	95% C.I OR	
GPs	0.998	NS	NS	NS
A/D	0.998	NS	NS	NS
Others	1.000	NS	NS	NS
Pediatrician	0.998	NS	NS	NS
20 – 30 years of practice	0.338	0.477	0.105	2.170
>30 years of practice	0.699	0.641	0.067	6.106
Aware of guidelines	0.060	0.268	0.068	1.059
Male	0.550	0.688	0.202	2.341
Urban	0.035	4.259	1.104	16.426

*fgAHs* first generation antihistamines

*p* value of model: 0.153

Internal medicine, <20 years of practice, not being aware of guidelines, female and rural location were references categories in the model

In approximately one quarter of patients (26.9%, SD 20.9), the first applied therapy was reported not to be successful (D/As 32.6%, GPs 28.6%, Internists 20.8% and Pediatricians 21.3%).

Only 12.9% of physicians used sgAHs in higher doses as second line treatment, and there is a statistically significant difference between D/A and GPs (51.9 vs. 7.7%; *p* < 0.001). Physicians stated that the use of sgAHs in normal or higher doses was successful in approximately 65% of CsU patients (Table 5). Physicians, who were familiar with the EAACI/GA^2^LEN/EDF/WAO guideline, were more likely to use higher doses of sgAHs (28.2 vs 8.2%, who don’t know the guidelines) (*p* < 0.001, Table 3). We could not find any statistical differences between physicians, who were aware of the EAACI/GA^2^LEN/EDF/WAO guideline and report the use of sgAHs at regular doses, and OCS as first line treatment compared to those who were not aware of the guideline (*p* > 0.05) (Table 3). With respect to third line therapies, the combination of sgAHs + leukotriene inhibitors is used by 7.5% of physicians, mostly by A/D (22.2%), and followed by pediatricians and GPs (7.1 and 5.1%) (*p* <0.01). The combination sgAHs + leukotriene inhibitor + H2 blocker was used by 8.0% of physicians, and D/As used it more frequently (25.9%, *p* < 0.01). Of the physicians who used these combinations, 55.9% reported to see good outcomes in their patients. Only 3.5% of physicians stated that they have experience with omalizumab and physicians who know the EAACI/GA^2^LEN/EDF/WAO guideline were more likely to use it (Table 3).

**Table 5:** Perception of satisfactory outcomes and side effects according to treatment

		Mean	SD
sgAH (normal doses)	% of patients with satisfactory outcomes	64.7	26.8
	% of patients without side effects	42.1	32.1
	% of patients with level of collaboration	73.6	30.4
sgAH (higher doses)	% of patients with satisfactory outcomes	62.3	30.7
	% of patients without side effects	45.2	32.2
	% of patients with level of collaboration	70.6	31.6
ssAH + AH2 blocker	% of patients with satisfactory outcomes	60.2	32.1
	% of patients without side effects	50.3	99.2
	% of patients with level of collaboration	83.5	99.9
sgAH + antileukotrienes	% of patients with satisfactory outcomes	54.7	31.5
	% of patients without side effects	52.0	32.7
	% of patients with level of collaboration	60.9	33.1
ssAH + AH2 blocker + antileukotrienes	% of patients with satisfactory outcomes	55.9	32.4
	% of patients without side effects	47.8	32.5
	% of patients with level of collaboration	72.6	30.3

*sgAHs* second generation antihistamines, *AH-2* anti-H2, *ALK* anti-leukotriene

### Adverse effects of therapy

Physicians reported that no adverse effects were observed in 42.1% of patients receiving regular doses of sgAHs and in 45.2% treated with higher doses of sgAHs. Elevating the doses of sgAHs didn’t appear to result in an increase in adverse effect rates (Table 5).

### Adherence and quality of life

The most frequent problems that physicians observed in their patients following CsU therapy, were reported to be in 38.6% adherence, 31.2% somnolence, 13.3% the cost of treatment and in 13.0% gastrointestinal symptoms. Physicians also reported that patients, who are resistant to treatment, are more vulnerable to have daily life problems, such as reduced QoL (40.9% of patients), social isolation (27.4%), and occupational disability (22.3%). Half of the physicians reported that their patients have an increased psychological burden as a consequence of the disease.

Approximately 50% of physicians responded that they need more time than usual for CsU patients and that the cost in medications and diagnostic tests for these patients is higher as compared to patients with other diseases. 62.1% of the physician reported referring patients to urticaria specialists.

## Discussion

This study shows that the knowledge of guideline recommendations in physicians who treat urticaria patients in Ecuador is low. The diagnostic workup and treatment of CsU patients is largely not in line with guideline recommendations in real life practice settings.

The EAACI/GA^2^LEN/EDF/WAO guideline for chronic urticaria has been recently updated [1], but the management of CsU varies among different parts of the world [1, 10–12]. Given the fact that this survey was previously used in Germany [7], we could compare results between the two regions and found that Ecuadorian physicians have lower awareness of the current guideline (33 vs 18%). Less than one quarter of GPs know the EAACI/GA^2^LEN/EDF/WAO guideline, but approximately two thirds of the D/As do. Physicians who know the guideline were aware of the importance of searching for the cause of CsU and this was found in approximately one third of patients. Interestingly specialist D/A, who report not to know the guidelines, still look for the etiology of CsU in their patients. (Fig. 1 a and b).

In the EAACI/GA^2^LEN/EDF/WAO guideline, only differential blood count and CRP or ESR are recommended as routine diagnostic tests for CsU patients [1]. Approximately half of the surveyed physicians don’t follow these recommendations. This could increase unnecessarily the costs of searching for CsU etiology.

Early studies suggested that almost 40% of the patients previously diagnosed with CsU had circulating autoantibodies, which might be implicated in the pathogenesis [13, 14].

A recent EAACI taskforce position paper proposed that the ‘gold standard’ for autoimmune chronic urticaria diagnosis should be a combination of a positive bioassay, positive auto reactivity and a positive immunoassay [15]. In Ecuador, we only have the possibility to do ASST (autologous serum skin test). Interestingly, 12.9% of participants use this test, in line with guideline recommendations. Notably, those physicians who were familiar with the current guidelines were significantly more likely to perform an ASST, similar to the results of the previous study [7]. Although the most common diagnostic test performed was the determination of total serum IgE (83.5%), ultimately it is not useful in the management of most chronic urticaria patients. We believe that, for a great number of GPs, chronic urticaria could be synonymous with allergy. Therefore, they perform this test and determine the total serum IgE.

The prevalence of parasitic infection in Ecuador is approximately 65%, especially soil-transmitted helminth species [16, 17]. In our country, parasites in the stool are not routinely looked for, despite of the high prevalence of parasitic infections. Because physicians are aware of the high prevalence of parasitic disorders in Ecuador, they prefer to treat CsU patients with antiparasitic drugs without looking for it.

Our study confirms that CsU severely affects patients’ quality of life. Acccording to the participating physicians of our study, more than 40% of patients have QoL impairment and 50% have psychological burden as a consequence of the disease. These results support the need to incorporate in the evaluation of CsU patients an objective diagnostic measure, a survey for example, to assess their QoL impairment and to rule out disorders such as depression and anxiety.

### Therapy of CsU

The EAACI/GA^2^LEN/EDF/WAO guideline for urticaria recommends the use of a regular dosed sgAHs as the first line therapy, followed by the use of sgAH in higher doses up to four times the regular dose, for those patients who do not respond to first line therapy [1, 10].

In our study, only one third of physicians reported to use regular doses of sgAHs as the first line treatment. Also, only 12.9% of physicians use sgAHs in higher doses and there is a statistic significant difference between D/A and GPs (Table 3). Physicians reported that 35% of the patients did not respond to this treatment. This contrasts with the results from the previous german study, where the majority of physicians reported to use regular doses and high doses of sgAHs as first and second line therapy, respectively.

Physicians still use fgAHs, particularly pediatricians; they probably consider this medication safe. After all, some practice parameters continue to recommend them [12]. In another study, hydroxyzine was the second most frequently prescribed drug, with no difference between dermatologists and allergists [18]. This could be the explanation why physicians, who are aware of the EAACI/GA^2^LEN/EDF/WAO guideline, are more likely to report using higher doses of fgAHs. Another possible explanation could be that fgAHs, in Ecuador, cost less than sgAHs.

The use of systemic steroids is not recommended as the first line therapy [1, 10, 12]; however it is still used by a meaningful number of physicians in our study. One possible explanation for the preference of steroids could be that these physicians have more experience and feel confident using them a rather than increasing the doses of sgAHs, as recommended in the guideline. Another explanation could be that they believe steroids relieve symptoms faster than other drugs. Interestingly, D/A reported having experience with oral steroids but less than half are not convinced that this therapy has been useful.

Increasing the dosage up to fourfold of modern second generation AH is a relatively new recommendation and probably physicians are not confident enough using them, because up-dosing antihistamines significantly improved control of pruritus but not of wheal number and there are weakness of the studies and the significant heterogeneity [19] or are afraid of possible adverse effects with the dosage increase.

On the other hand, systemic steroids are cheaper than the fourfold dosage of sgAHs and this difference in costs could influence the physician’s decision. We believe all this could explain the low use of higher doses of sgAHs in our study. Indeed, coverage and payment for healthcare may play an important role in our country. As previously discussed, costs vary among drugs and this may influence patient and physicians’ choices. Public hospitals may not cover third line therapy in our country, thereby obstructing with physicians’ treatment and management.

Differing to the previous German study, we could not find a difference between physicians aware vs not aware of the EAACI/GA^2^LEN/EDF/WAO guideline regarding the likelihood of using regular dosed fgAHs or systemic steroids as a first-and/or second-line treatment. In Germany, physicians familiar with the guideline were less likely to use them [7].

Similar to the German study, the participants of our study reported that high dosing of sgAHs is effective in a higher percentage of patients when compared to regular doses [7]. But only few physicians use this approach in real life as previously discussed.

As for third line therapy, the use of the combination of sgAHs and leukotriene antagonists or H2 blockers was reported only by few physicians, and one third of them have satisfactory outcomes in their patients (Table 5). Pediatricians used montelukast more frequently than other groups, probably because they are familiar with this drug from treating asthma and rhinitis.

In our study only around 15% of physicians have experience with immunosuppressive drugs and omalizumab, D/A being the most experienced group. It is interesting that physicians familiar with the EAACI/GA^2^LEN/EDF/WAO guideline prescribed omalizumab more frequently. Omalizumab was approved for CsU in 2014 in our country. In another study of our group about omalizumab for CsU, we found that 77% of patients had a complete or partial response after treatment of 3 months; however, 65.4% of patients did not complete 3 months of treatment, likely owing to the cost of omalizumab and it not being reimbursed by health insurance programs [20].

### Limitations and strengths of the study

The validity and reliability of the original version of the survey had not been reported, but the selection of the items was developed using a rigorous method [9]. The present study did not intend to validate the questionnaire. Consequently, face validity and reliability need to be established in a future study. Also, the survey-based design may present reporting and recall bias.

All results are specific for Ecuador and cannot be generalized to other countries in the region given the different healthcare systems and government funding, changing probably the management of CsU between countries.

In our country, patients can be attended at public hospitals, such as hospitals from the Social Security System or the Ministry of Health, or private practices for consultation. Public hospitals tend to have less time per patient, and in the case of chronic urticaria, it is well-known that physicians need more time with these patients for a good consultation (approx.30 min). Consultations of less than 10 min are unacceptable and do not allow for adequate patient care [21]. Studies suggest approx. 18–20 min per patient should be available to satisfy the patient and accomplish quality standards of medical consultation and patient care [22, 23].

One group of the surveyed physicians was attending medical meetings and conferences; their medical knowledge is more likely to be updated than the surveyed group of physicians who do not attend continuing medical education meetings.

Also we cannot compare one to one our results with the previous German study, since the distribution of specialties were different. In the German study, most of the surveyed physicians were dermatologists (43%) and in our study GPs (67.6%). This could explain some differences in the findings. Nevertheless, to the best of our knowledge, this is the first study that explores CsU management in Latin-America and compares it with Europe. Our results suggest that disparities in knowledge between physicians from different countries could influence the management of CsU. In a previous study, we reported differences between Latin American and European physicians in the management of asthma and the patient-physician relationship [24]. Another strength of our study is the high rate of participants (*n* = 740), larger than the calculated sample size. Also, because of the high rate of participating GPs in our study, we could identify a relevant lack of knowledge in CsU management among these physicians. The increased diffusion of the current guidelines could improve the knowledge of diagnostic and therapeutic management for CsU patients and could lead to improved outcomes and better patient care.

## Conclusion

This study is the first to describe guideline knowledge and the real life management of CsU among physicians in a Latin-American country. Of note, it showed a low awareness of the current EAACI/GA^2^LEN/EDF/WAO guideline; although there is significant number of patients with CsU. It also showed that only a limited number of physicians are using third level medication when needed.

Our results suggest that differences in knowledge between physicians from different countries could influence the management of CsU and this should be considered and confirmed in future studies.

We believe that despite increasing efforts to disseminate knowledge and awareness of chronic urticaria, important information about management does not reach the GPs and specialists. Awareness and knowledge of chronic urticaria needs to increase especially among GPs, because they are often the first physicians to be consulted by patients, and the low knowledge of guidelines could influence CsU control and treatment, delaying the consultation by the specialist.

## Additional files

Additional file 1: Figure S1. Awareness of the EAACI/GA2LEN/EDF/WAO guidelines according to specialty in percentage. (JPEG 87 kb)

Additional file 2: Figure S2. Awareness of other guidelines about management of CsU among all physicians in percentage. (JPEG 89 kb)

## Abbreviations

ASST: Autologous serum skin test
CsU: Chronic spontaneous urticaria
CU: Chronic urticaria
D/A: Dermatologist/Allergist
fgAHS: First generation H1-antihistamines
GP: General physician
OCS: Oral corticosteroids
QoL: Quality if life
sgAHS: Second generation H1-antihistamines
