# Supplementary figures and images for: Knowledge and management of chronic spontaneous urticaria in Latin America: a cross-sectional study in Ecuador
Source: World Allergy Organ J. 2017 May 23;10(1):21. doi: 10.1186/s40413-017-0150-7 (PMC5440895; doi:10.1186/s40413-017-0150-7)

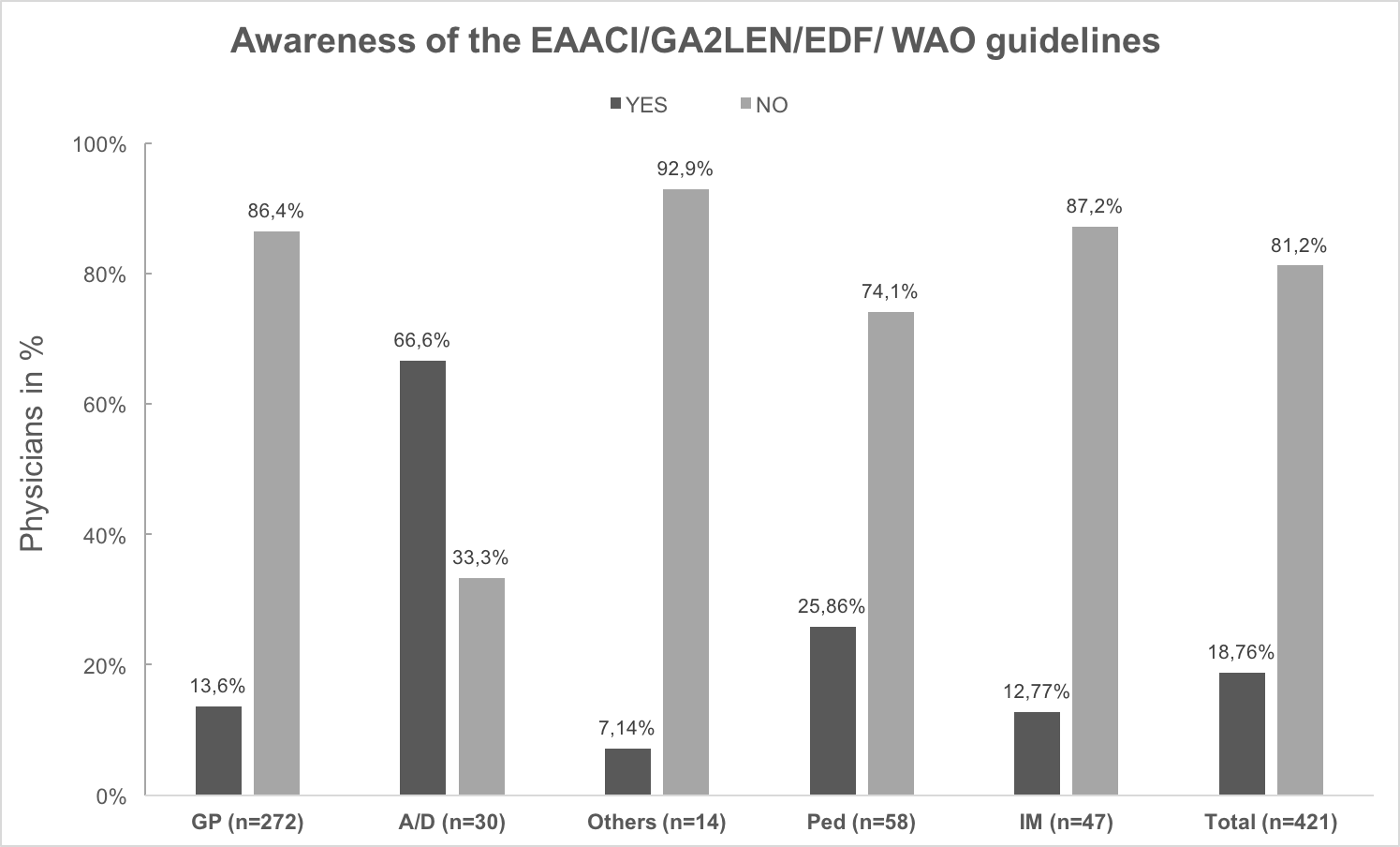

Supplement: Supplementary file 1 — Awareness of the EAACI/GA2LEN/EDF/WAO guidelines according to specialty in percentage. (JPEG 87 kb) [file 40413_2017_150_MOESM1_ESM.jpeg]

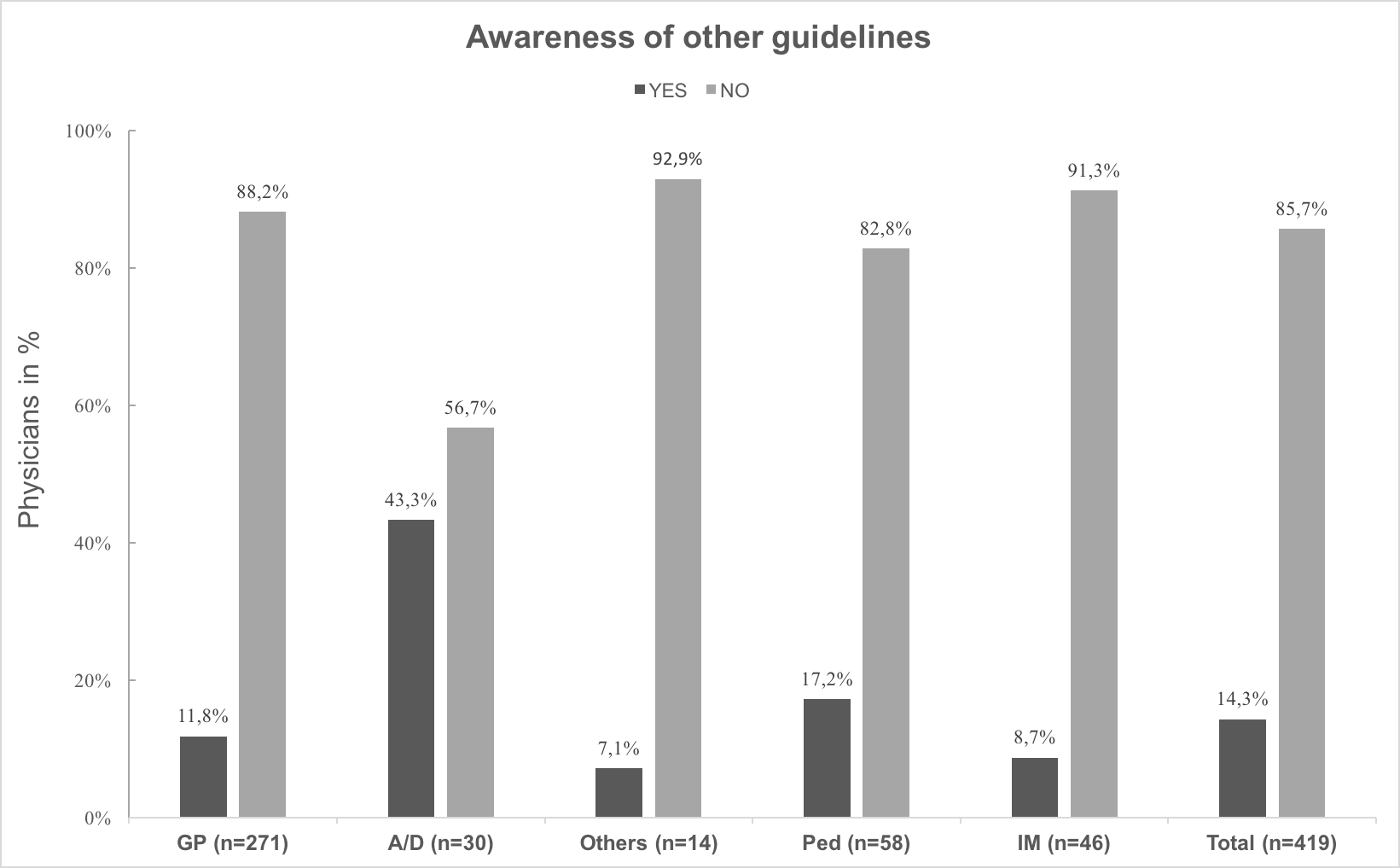

Supplement: Supplementary file 2 — Awareness of other guidelines about management of CsU among all physicians in percentage. (JPEG 89 kb) [file 40413_2017_150_MOESM2_ESM.jpeg]
